# Influence of Tantalum Addition on the Corrosion Passivation of Titanium-Zirconium Alloy in Simulated Body Fluid

**DOI:** 10.3390/ma15248812

**Published:** 2022-12-09

**Authors:** El-Sayed M. Sherif, Yassir A. Bahri, Hamad F. Alharbi, Muhammad Farzik Ijaz, Ibrahim A. Alnaser

**Affiliations:** 1 Centre of Excellence for Research in Engineering Materials (CEREM), King Saud University, P.O. Box 800, Riyadh 11421, Saudi Arabia; 2 Mechanical Engineering Department, Collage of Engineering, King Saud University, P.O. Box 800, Riyadh 11421, Saudi Arabia

**Keywords:** Ti-base alloys, Ta addition, corrosion passivation, simulated body fluid, electrochemical techniques, spectroscopic analysis

## Abstract

Ti-15%Zr alloy and Ti-15%Zr-2%Ta alloy were fabricated to be used in biomedical applications. The corrosion of these two alloys after being immersed in simulated body fluid for 1 h and 72 h was investigated. Different electrochemical methods, including polarization, impedance, and chronoamperometric current with time at 400 mV were employed. Also, the surface morphology and the compositions of its formed film were reported by the use of scanning electron microscope and energy dispersive X-ray. Based on the collected results, the presence of 2%Ta in the Ti-Zr alloy passivated its corrosion by minimizing its corrosion rate. The polarization curves revealed that adding Ta within the alloy increases the corrosion resistance as was confirmed by the impedance spectroscopy and current time data. The change of current versus time proved that the addition of Ta reduces the absolute current even at high anodic potential, 400 mV. The results of both electrochemical and spectroscopic methods indicated that pitting corrosion does not occur for both Ti-Zr and Ti-Zr-Ta alloys, even after their immersion in SBF solutions for 72 h.

## 1. Introduction

The current demand for biomedical implants has shaped the need for high-quality materials to meet such a market. Statistically, it is estimated that approximately 1.0 million people undergo hip replacements annually, which further establishes the significant use of biomedical equipment on a global scale [1,2,3,4,5,6,7]. Ti-base alloys have been employing in automotive, aerospace, and biomedical employments for their excellent mechanical strength, high corrosion resistance, and good biocompatibility [4,6]. Some of the Ti-based alloys still present several mechanical performance challenges [7]. In addition, even though titanium is well known for its biocompatibility, Ti–6Al–4V is the most employed alloy for biomedical applications. This alloy contains aluminum as well as vanadium, which raises worries that ions from these metals could have a negative effect on the application [8]. It has been claimed [9,10] that Ti-6Al-4V alloy causes toxic reactions within the human body as a result of releasing of Al^+3^ and V^+5^ as free radicals, which interact with the bloodstream. This release of these cations leads to a possible risk of Alzheimer’s disease within the patients [11,12]. Because of this, it is desirable to substitute these metals for other alloying elements.

Zirconium (Zr) and tantalum (Ta) have been proposed as more stable non-toxic titanium alloys and biocompatible alternatives to these metals [13,14,15]. Zr has excellent mechanical properties, as well as a remarkable corrosion resistance, which makes it a prefect substance for biomedical implants. In addition, Ta is non-reactive with bodily fluids and is utilized in the production of surgical instruments. In addition, Ta is likewise non-irritating to the body, and it is utilized in the manufacture of surgical sutures and implants such as prosthetic joints and cranial plates, among other things. A protective oxide film formed on the outer surface of such alloys gives it excellent in-vivo performance. The formation of this layer reduces the number of the released cations into the biological medium and aids in the osseointegration process. Given that the implant surface is the only location in which living tissues come into contact, the properties of the surface coating are of particular importance [16]. It is possible to improve the resistance to corrosion and biocompatibility of permanent implant materials by altering their surface properties. Due to the excellent wear resistance of Zr, commercially available Zr knee implants with surface oxide coatings thicknesses ranging from 2–5 µm are used [17].

This study investigated the effect of adding 2% Ta on the passivation of corrosion of Ti-15Zr alloy in the simulated body fluid (SBF) solution. The SBF was selected since its effect on Ti-Zr alloy is similar to that of blood. Various techniques to evaluate the corrosion; these are the cyclic polarization, impedance, and the change of current vs. time were used for alloys after their exposure for 1 h and 72 h in the SBF solution. Scanning electron microspectroscopy and X-ray spectroscopy investigations were also conducted on the surface of the corroded samples.

## 2. Materials and Methods

The powders of Ti (99.9 purity and 5–7 μm particle size), Zr (99% purity and 10 μm particle size), and Ta (99.9% purity and 10 μm particle size) were supplied by Nanochemazone, Canada. Ally powders were mixed (in weight percentage) in a high-energy ball mill. To ensure the homogenous distribution for all powders, the mixing rate was 150 rpm for 30 min. The electrolyte was a simulated body fluid (SBF, Gibco^®^ by Life technologies, New Delhi, India), which is a mixture of the following chemical substances, NaCl, KCl, Na_2_SO_4_, NaHCO_3_, K_2_HPO_4,_ MgCl_2_.6H_2_O, and CaCl_2_ [18]. The manufacturing of the Ti-Zr alloy and Ti-Zr-Ta alloy was carried out with the same reported procedures in the previous study [19].

A three electrodes electrochemical cell that widen for 150 mL SBF was employed. A computer-controlled Autolab Potentiostat/Galvanostat (purchased from Metrohm, Amsterdam, The Netherlands) was utilized. The preparation of the working electrodes, which were the fabricated alloys, was carried out as in the published work [17,19]. Also, the reference and counter electrodes were the same ones that were used in the reported study [19]. The polarization curves were obtained by scanning the potential from −1000 to +400 mV at a scan rate of 2.0 mV/s. The impedance measurement experiments were collected from the corrosion potential within a frequency range between 100,000 Hz and 0.10 Hz and the data were fitted using the ZSimpWin v3.1 software [20,21,22]. The chronoamperometric current-time curves were obtained after stepping the potential at an anodic value in the SBF solution for 60 min of the actual measurement from applying the anodic constant potential, 400 mV. All experiments were performed at room temperature after 1 h and 72 h exposure to the test solution. The surface morphology was taken using a JEOL microscopy model JSM-7400F (SEM, Tokyo, Japan) and the EDX analyses were also investigated using an attached unit to the microscope.

## 3. Results and Discussion

### 3.1. Potentiodynamic Cyclic Polarization (PCP) Data

**Figure 1:**
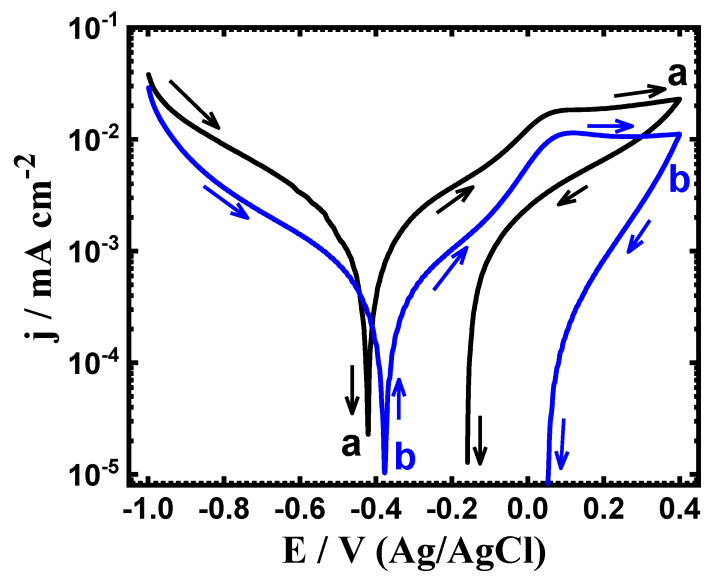
PCP curves of (**a**) Ti-Zr alloy and (**b**) Ti-Zr-Ta alloy immersed in SBF for 1.0 h.

**Figure 2:**
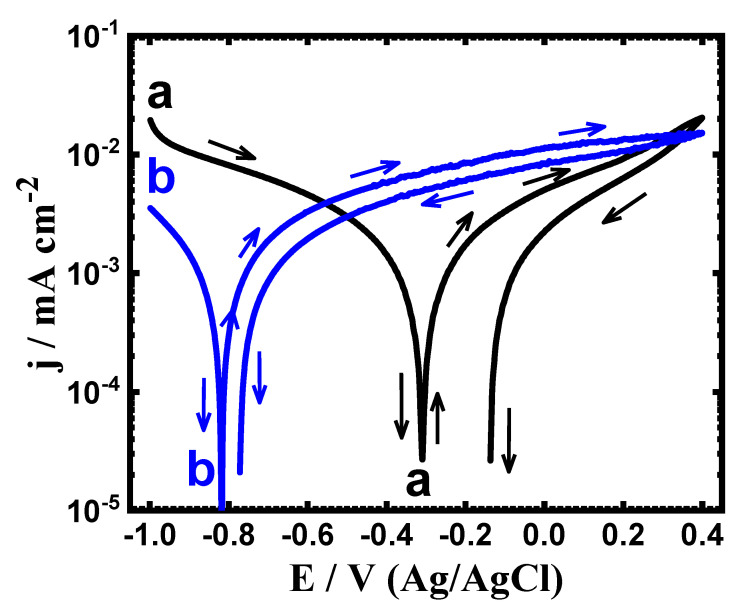
PCP curves of (**a**) Ti-Zr alloy and (**b**) Ti-Zr-Ta alloy immersed in SBF for 72 h.

The PCP experiments were conducted to determine the effects of Ta additions on the corrosion behavior of the Ti-Zr alloy in the SBF solution at two exposure periods of time. Figure 1 presents the PCP plots for (a) Ti-Zr alloy and (b) Ti-Zr-Ta alloy after being immersed in the SBF solution for 1 h. Same procedures were also carried out after 72 h immersion and the curves of the PCP are displayed in Figure 2. The values predicted from those polarization figures are registered in Table 1. These symbols are the cathodic (*β_c_*) and anodic (*β_a_*) Tafel slopes, respectively. The corrosion potential (*E_Corr_*), the corrosion current (*j_Corr_*), the polarization resistance (*R_P_*), and the corrosion rate (*R_Corr_*). The *β_c_*, *β_a_*, *j_Corr_* and *E_Corr_* values were obtained from the plotted curves as has been reported in the earlier work [20,21,22]. The *R_P_* values as well as the *R_Corr_* values were calculated using the following relations, respectively [23,24,25,26,27]:

(1) $$R_{P}=\frac{1}{j_{Corr}}\;\left(\frac{\beta_{c}\cdot \beta_{a}}{2.3\left(\beta_{c}+\beta_{a}\right)}\right)\;$$

(2) $$R_{Corr}=j_{Corr}\left(\frac{k\cdot E_{W}}{d\cdot A}\right)$$

Here, *k* = 3272 mm (amp^−1^ cm^−1^ year^−1^), *E_W_* = equivalent weight, d is the density, and A is the area. Figure 1 and Figure 2 illustrated that the cathodic current tends to decrease towards the minimum value (where, *j_Corr_* and *E_Corr_* are calculated) as a result of the oxygen reduction reaction as per these reactions [27,28,29];

2H_2_O + O_2_ + 4e^−^ = 4OH^−^ (3)

(4) $$\frac{1}{2}O+H_{2}O+2e^{-}=2OH^{-}$$

For Ti-15Zr alloy and Ti-15Zr-2Ta alloy, the anodic reaction begins with increasing the current values with potential scan in the positive direction. It is also observed that the Ti-15Zr’s anodic current is higher than that of Ti-15Zr-2Ta’s anodic current at the same potential value. This means that Ti-15Zr-2Ta alloy has higher resistance to corrosion than that the Ti-15Zr alloy has. Scanning the potential in the backward direction, both Ti-15Zr alloy and Ti-15Zr-2Ta alloy provided lower current values than those obtained from the forward direction. The disappearance of hysteresis loops with both alloys indicates that the pitting attack does not occur at this condition. As the immersion period is prolonged to 72 h (Figure 2), the obtained PCP curves were similar to those recorded after only 1 h exposure (Figure 1), but with lower *j_Corr_* and *E_Corr_* values. These results are corroborated by the values recorded in Table 1, where *R_Corr_* and *j_Corr_* are smaller, whereas *R_P_* is higher for the Ti-15Zr-2Ta alloy, and this effect occurs with increasing exposure time to 72 h. Therefore, the PCP results confirm that adding 2%Ta to Ti-15Zr increases its corrosion resistance when exposed to SBF, and that this effect increases with increased exposure time. In fact, it is more than likely that the formations of TiO_2_, ZrO_2_ and TaO_2_ mixed oxides account for higher resistance to corrosion as well as less corrosion rates in Ti-15Zr-2Ta alloys. Meanwhile, due to the formation of TiO_2_ and ZrO_2_ in case of the Ti-15Zr alloy, it records a lower *R_P_* value and higher *R_Corr_* value than those for Ti-15Zr-2Ta alloy.

### 3.2. Electrochemical Impedance Spectroscopy (EIS) Measurements

**Figure 3:**
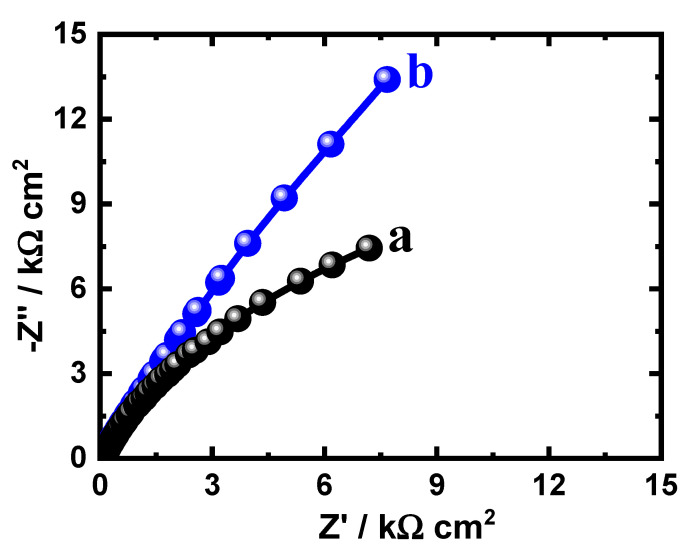
Nyquist plots for (**a**) Ti-Zr alloy and (**b**) Ti-Zr-Ta alloy after their immersion in SBF solution for 1.0 h.

**Figure 4:**
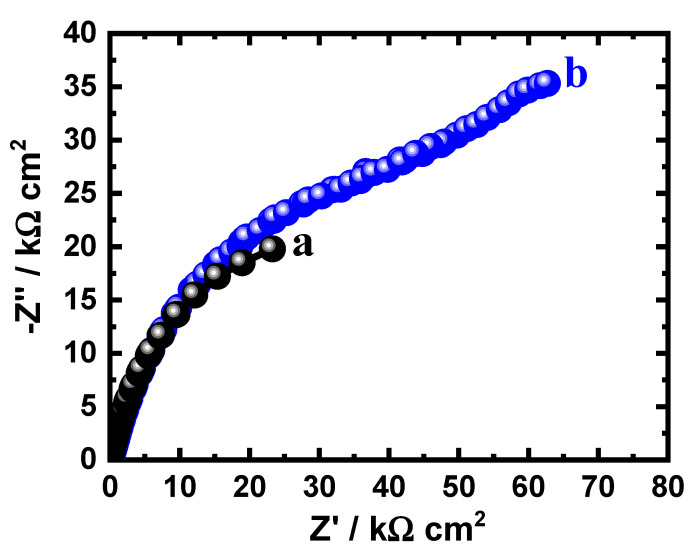
Nyquist plots for (**a**) Ti-Zr alloy and (**b**) Ti-Zr-Ta alloy after their immersion in SBF solution for 72 h.

**Figure 5:**
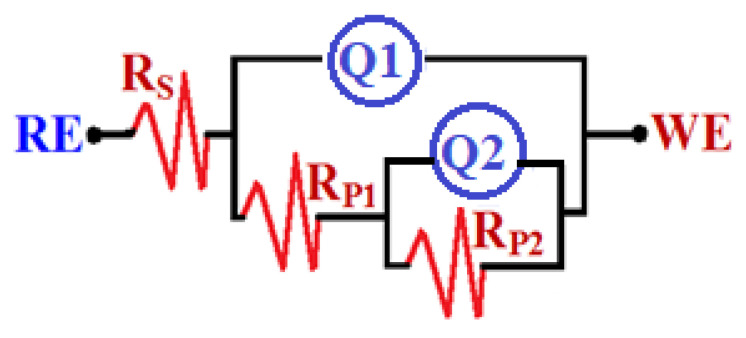
The R(QR(QR)) equivalent circuit model.

The data obtained from EIS provide an explanation for the reactions of the electron transfer at the interface for the alloys and SBF solution. EIS technique has been successfully employed for reporting the corrosion and passivation phenomena under various circumstances [26,27]. Exposure to SBF solution for 1.0 h, the Nyquist plots for (a) Ti-15Zr alloy and (b) Ti-15Zr-2Ta alloy are presented in Figure 3. In addition, similar plots were also collected after 72 h at the same condition, and the spectra are depicted in Figure 4. These data were best fitted to the circuit that is displayed in Figure 5. This circuit have different impedance elements. Here, the solution resistance (*R_S_*), two different constant phase elements (*Q*_1_ and *Q*_2_), and two polarization resistances (*R_P_*_1_ and *R_P_*_2_). Table 2 presents these elements and summarizes the values of the EIS elements. In order to make sure the chosen circuit that was used here, other studies have reported the same circuit for other materials [30,31,32].

It has been proposed that the existence of *CPEs* in the capacitive circuit enables us to estimate the deviations from ideality. Here, *Q*_1_ with n values varying from 0.75 to 0.86 exemplify double layer capacitors with some pores to reduce the chare of the alloys’ surfaces. Furthermore, *Q*_2_ represents another double layer capacitor for its n values in the range from 0.61 and 1.0, which increases the concept that the passivation of the tested alloys in SBF solution is achieved, while the dissolution is not favorable. Also, *R_P_*_1_ signifies that polarization resistance exists between the surface Ti-Zr alloy and Ti-Zr-Ta alloy and the layer formed on their surfaces. Additionally, *R_P_*_2_ refers to the resistance at the interface between the alloys’ formed surface layer and electrolyte.

The EIS spectra of Figure 3 and Figure 4 along with the listed elements in Table 2 show that Ta rises the Ti-Zr-Ta alloy’s resistance to corrosion. This is indicated by the diameter of the obtained semicircle that is much wider than the one of the circle of Ti-Zr alloy. This was also enhanced via the higher values of the obtained polarization resistance, *R_P_*_1_ and R_P2_, as represented in Table 2. Another proof of the increase of corrosion resistance was predicted from the values of *Y_Q_*_1_ and *Y_Q_*_2_ as it decreases in the presence of Ta. Immersion for 72 h also has a great impact on enhancing the values of the resistance to corrosion for both Ti-Zr alloy and Ti-Zr-Ta alloy. This was revealed by increasing the diameter of the Nyquist plot (Figure 4) and the higher *R_P_*_1_ and *R_P_*_2_ values, in addition to the lower *Y_Q_*_1_ and *Y_Q_*_2_ values (Table 2).

**Figure 6:**
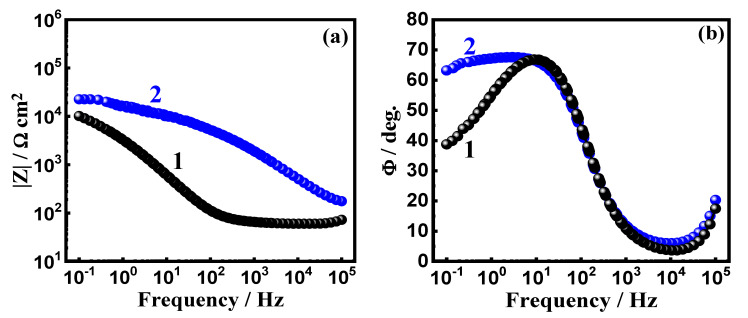
(**a**) Bode *|Z|* and (**b**) Bode *Φ* plots for (1) Ti-Zr alloy and (2) Ti-Zr-Ta alloy that were immersed in SBF for 1 h.

**Figure 7:**
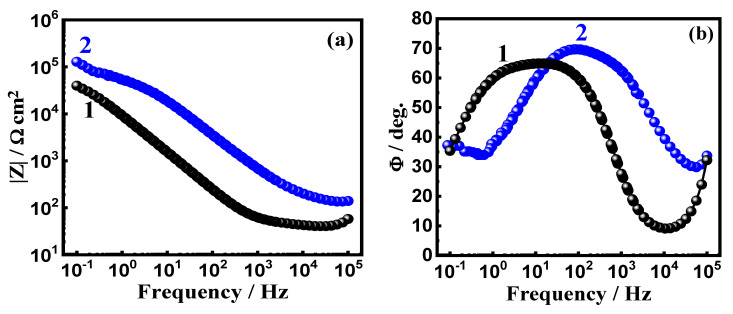
(**a**) Bode *|Z|* and (**b**) Bode *Φ* plots for (1) Ti-Zr alloy and (2) Ti-Zr-Ta alloy that were immersed in SBF for 72 h.

Bode plots of (a) impedance of the interface (*|Z|*) and (b) degree of the phase angle (*Φ*) for (1) Ti-Zr alloy and (2) Ti-Zr-Ta alloy that were exposed to SBF for 1 h are presented in Figure 6. Similar plots were also taken for these alloys after their immersion in SBF for 72 h, as seen in Figure 7. As seen from Figure 6, the presence of Ta enhances *|Z|* values, and the highest value of *Φ*, particularly at the low frequency region. This indicates that the passivity of the Ti-Zr alloy when Ta is present increased. As compared to the values of *|Z|* and *Φ* after 1 h immersion, the plots of Figure 7 indicate that extending the time of exposure to 72 h further increases both *|Z|* and the highest heights of *Φ*, which is clearer at the low frequency values. The high corrosion resistances of both Ti-Zr and Ti-Zr-Ta alloys increase remarkably with expanded exposure time to SBF solution. The EIS results along with the PCP data confirm that adding 2% Ta greatly improves the Ti-Zr alloy’s resistance to corrosion, and this is remarkably enhanced at the longer immersion period.

Bode plots of the (a) impedance of the interface (*|Z|*) and (b) degree of the phase angle (*Φ*) for Ti-Zr alloy (1) and Ti-Zr-Ta alloy (2) after being exposed to SBF for 1 h are presented in Figure 6. The same plots were also taken for these alloys after their immersion in SBF for 72 h, as seen in Figure 7. As seen from Figure 6, the presence of Ta enhances *|Z|* and the highest *Φ* values, particularly at the low frequency region. This indicates that the passivity of the alloy when Ta is present increased. As compared to the values of *|Z|* and *Φ* after 1 h immersion, the plots of Figure 7 indicate that extending the time of exposure to 72 h further increases both *|Z|* and *Φ*, which is clearer at the low frequency values. The high corrosion resistances of both Ti-Zr and Ti-Zr-Ta alloys increase remarkably with the exposure time to SBF solution. The EIS results along with the PCP data confirm that adding 2% Ta greatly improves the Ti-Zr alloy’s resistance to corrosion and this is enhanced remarkably at the longer immersion period.

### 3.3. Chronoamperometric Current-Time (CCT) Measurements

**Figure 8:**
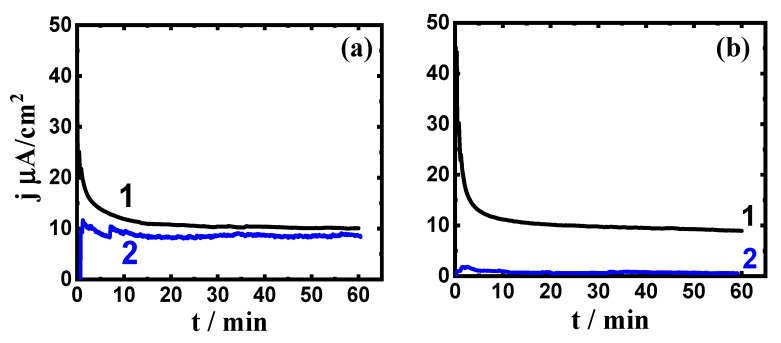
The CCT plots for (1) Ti-Zr alloy and (2) Ti-Zr-Ta alloy after (**a**) 1 h and (**b**) 72 h exposure to SBF solution, the applied potential was 400 mV, respectively.

The CCT plots at 400 mV were collected to report the influence of Ta on the resistance to corrosion of Ti-Zr. In particular, pitting corrosion occurs, where the CCT technique has been widely employed to report similar cases [33,34,35,36]. Figure 8 depicts the curves of the current variations vs. time of (1) Ti-Zr alloy and (2) Ti-Zr-Ta alloy over (a) 1 h and (b) 72 h in SBF, respectively. Here, the current of Ti –Zr alloy (curve 1) shows the highest values at the first moments measurement, whether the alloy is exposed for 1.0 h or 72 h. In this case, the lessening of current results from the formation of oxide film, which gives the surface an immunity against the attack of the corrosive SBF solution. Expanding the time of the potential application for ≥20 min produced a slight decrease of the current that in turn stabilizes the formed oxide layer to passivate the surface [18]. This again provides another confirmation that pitting corrosion does not take place for the alloy and general corrosion is at its minimum. The lower current values in the presence of Ta do not only passivate the surface and enhance its resistance to general corrosion but also decrease the severity of pitting corrosion. It is worth noting that expanding the time to 72 h leads to an increase in corrosion resistance.

### 3.4. SEM and EDX Analyses

**Figure 9:**
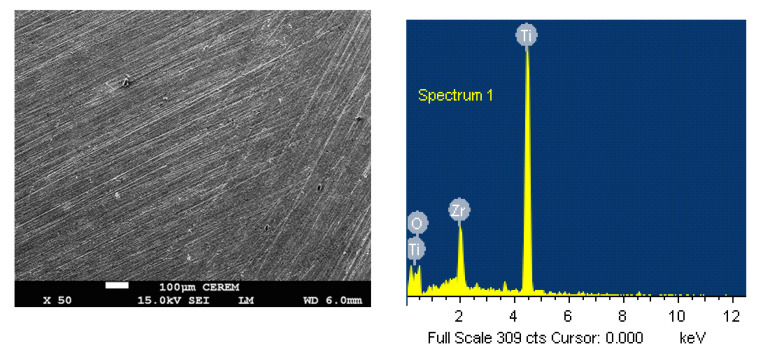
The SEM and EDX for Ti-Zr alloy after its exposure for 3 days in SBF before stepping the potential to 400 mV.

The effect of adding 2% Ta on the surface morphology of Ti-Zr alloy after being immersed in SBF for 3 days and stepping a 400 mV as a constant anodic potential for an hour was determined by scanning electron microscope (SEM) images. Figure 9 shows the SEM image and the energy dispersive X-ray (EDX) spectra for Ti-15Zr under the aforementioned conditions. The SEM image of Figure 9 displays that the Ti-15Zr alloy surface does not show any deep pits with the possibility of formation of some corrosion products. The chemical components found on that surface for the Ti-15Zr sample that is shown in the EDX were only Ti, Zr, and O, with proportional weights of 64.19% (Ti), 22.05% (O), and 13.7% (Zr) respectively, and atomic of 46.67% (Ti), 45.86% (O), and 7.47% (Zr). According to the collected results, the detected O content indicates that the formed surface film on the alloy probably is a mixture of TiO_2_ and ZrO_2_, and thus the passivation of Ti-Zr alloy in SBF solution is increased, and pitting corrosion is prevented from taking place [19].

**Figure 10:**
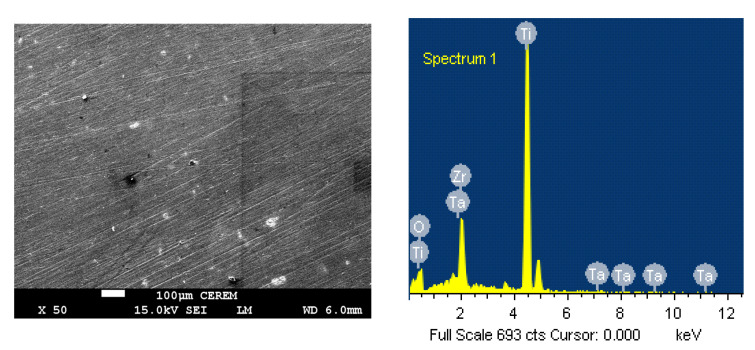
The SEM and EDX for Ti-Zr-Ta alloy after its exposure for 3 days in SBF before *stepping* the potential to 400 mV.

Figure 10 displays (a) SEM and (b) EDX analyses for Ti-15Zr-2Ta alloy that were taken under the same procedures considered for Ti-15Zr alloy. It is clear that the surface of Ti-15Zr-2Ta alloy is more similar to the surface of Ti-15Zr and that no clear pittings appear. The EDX spectrum confirmed the presence in weight % of 66.53% Ti, 14.99% Zr, 1.56% Ta, and 16.91% O, which is equivalent to the atomic % of 53.03% Ti, 6.27% Zr, 0.33% Ta, and 40.36% O, on the surface of the Ti-Zr-Ta alloy. The presence of high oxygen content again suggests a thick oxide layer that consists of TiO_2_, ZrO_2_, and TaO_2_ is formed on the surface. The surface morphology using SEM and the elemental analysis using EDX tools are thus consistent with the electrochemical data, which proved the influence of 2% Ta addition, namely that it remarkably increases the resistance to corrosion of the titanium alloy. As compared to the reported research on pure Ti [19], CP-Ti [37], TaC nanocrystalline coating [38], Ti-7.5%Mo [39], and also compared to our previous works on Ti-6%Alalloy, Ti-6%Al-4%V alloy [35], and Ti-6Al-xV alloys [36], the fabricated Ti-Zr-Ta here has low corrosion rate and excellent corrosion resistance. Moreover, compared to the Zhou et al. [5] who reported the corrosion resistance, wear resistance, and biocompatibility of Ti-10Ta alloy, Ti-30Ta alloy, and Ti-70Ta alloy, the presence of Zr and Ta within Ti in our tested Ti-Zr and Ti-Zr-Ta alloys has greatly improved the corrosion resistance.

## 4. Conclusions

The passivation of corrosion of Ti-15%Zr alloy and Ti-15%Zr-2%Ta alloy, which were fabricated from their milled powders using HFHIF, after being immersed for in SBF solution 1 h and 72 h, were investigated. The PCP results indicated that the addition of 2% Ta decreased the corrosion of Ti-15Zr alloy by decreasing its corrosion current and thus its corrosion rate, which increases its corrosion resistance. EIS Nyquist and Bode plots also confirmed that the presence of 2% Ta minimizes corrosion of Ti-15Zr alloy through increasing the diameter of the obtained semicircle and the values of the impedance of the interface as well as the maximum of the degree of the phase angle. By comparing Ti-15%Zr-2%Ta alloy with Ti-15%Zr, CCT measurements demonstrated that the absolute currents were lower when Ta was present in the alloy. The increase of time of immersion for the tested alloys in SBF solution has been found to reduce their corrosion via the thickening of a mixture of oxide layers. The surface investigations confirmed that Ta passivates the corrosion of Ti-Zr alloy via the formation of TiO_2_, ZrO_2_ and TaO_2_. All results support the proposal that the investigated alloys can provide a great benefit in employment in biomedical applications. Compared to the other reported pure Ti and Ti-base alloys; i.e., Ti-6Al, Ti-6Al-2V, Ti-6Al-4V, Ti-6Al-6V, Ti-6Al-8V, the manufactured Ti-Zr and Ti-Zr-Ta alloys produce lower corrosion rate and higher corrosion resistance.

## Figures and Tables

**Table 1:** Parameters estimated from the PCP curves.

Alloy	*β_c_* (V dec^−1^)	E_Corr_ (V)	*β_a_* (V dec^−1^)	*j_Corr_* (mA/cm^2^)	*R_P_* (kΩ cm^2^)	*R_Corr_* (mmy^−1^)
Ti−Zr (1.0 h)	190	160	425	0.55	68.66	6.40 × 10^−3^
Ti−Zr-Ta (1.0 h)	140	150	365	0.35	89.96	4.09 × 10^−3^
Ti−Zr (72 h)	160	180	315	0.45	81.84	5.23 × 10^−3^
Ti−Zr-Ta (72 h)	150	170	300	0.30	115.49	3.49 × 10^−3^

**Table 2:** EIS parameters represented in Figure 5.

Alloy	*R_S_*_/_Ω cm^2^	*Q*_1_		*R_P_*_1_/Ω cm^2^	*Q_2_*		*R_P_*_2_/Ω cm^2^
		*Y_Q_*_1_/F cm^−2^	n		*Y_Q_*_2_/F cm^−2^	n	
Ti−Zr (1.0 h)	60.3	0.0418	0.78	4360	0.0093	1.00	5512
Ti−Zr-Ta (1.0 h)	72.4	0.0411	0.86	6869	0.0066	0.61	8400
Ti−Zr (72 h)	62.5	0.0319	0.80	10,122	0.0051	0.81	20,653
Ti−Zr-Ta (72 h)	122.5	0.0212	0.75	12,526	0.0012	0.90	26,850

## Data Availability

Not applicable.
